# Professional Longevity as a Problem of the Value-Semantic Regulation of Teacher Activity

**DOI:** 10.3390/bs9120151

**Published:** 2019-12-10

**Authors:** Natalia G. Zotova, Svetlana A. Peredelskaya, Mikhail Yu. Tikhomirov

**Affiliations:** Department of Psychological and Teacher Training Education, Volgograd State Pedagogical University, Volgograd 400066, Russia; peredelskaya@gmail.com (S.A.P.); tcmich@mail.ru (M.Y.T.)

**Keywords:** value-meaning regulation, teacher, professional longevity, self-efficacy, burn-out, acme, life meaning orientations

## Abstract

The aim of this research is to establish proper external and internal conditions for conducting pedagogical activity that reveal the value basis of professional education. The research justifies the need to analyse the guidelines of a person’s professional self-identity, contents of professional activity motivation, peculiarities of maintaining the mental and physical health of a person, and the capacity of an educational institution’s organisational culture. A number of indicators of value mechanisms of a teacher’s professional longevity is revealed. A diagnostic program of the project, aimed at studying the value content of a teacher’s behaviour strategies during the process of professional activity, was designed. The content of the hypothesis is connected with the assumption of value-semantic inter-conditionality of professional longevity. The research revealed significant interrelations of professional longevity with some psychological factors of value-semantic nature. Analysis of the organisational culture of an educational institution as a factor providing (preventing) the professional longevity of a teacher was carried out. The dominant tendency of describing the interconnection between an educational institution’s organisational culture and the level of emotional burnout syndrome of teachers working within certain cultural models is revealed.

## 1. Introduction

The problem of productive professional longevity has turned out to be relevant at the present stage of society’s development. National projects in the area of “Education” reflect the development strategies of educational systems of any state, and these are not only prospects for the use of various forms of advanced training for adults. In the life of a person at this age, it is important to preserve psychophysical capabilities as well as to achieve professionally significant results. Of the respondents who took part in our study, 75% expressed the desire to extend the period of professional longevity. Among the professed reasons were the need to maintain a certain social status, financial status, and a habitual lifestyle;the possibility of maintaining comfortable conditions of work, and taking a rest. However, the average age of teachers in Russia is approaching 47–48 years (pre-retirement age), and mental and physical health deteriorates with age. In addition, the professional activity of a teacher is characterised by a high level of mental and physical stress, which leads to the loss of vital energy, emotional distress, and burnout. The saturation of the profession with crisis factors is supplemented by existential problems of a person’s stage of maturity, which is invariably accompanied by a decrease of social and cognitive activity.

Thus, the theory of the psychosocial stages of a person’s development [1] states that the main problem of the age of maturity (from 26 to 64 years old) is the choice between productivity and inertia. Productivity is embodied in the form of the older generation’s concern about those who will replace them. Inertia is expressed in the self-absorption of a person, aimed at the realisation of his or her interests. This phenomenon—the “old age crisis”—is expressed in a sense of hopelessness and meaninglessness of life.

This problem has been viewed from different angles;for example, from the point of view of functional approach measurements, studies, training of mental functions, special states, psychological characteristics of a person, and the abilities of a teacher. The acmeological approach considers the analysis of the natural possibilities of a person at different stages of an individual’s development and the achievement of “acme”—the highest point of professional development [2]. In modern Russian science, among relevant areas of the problems of personal performance research, the following can be mentioned: “vector modelling of personal development environments” [3], “intellectual initiative” [4], the nature of “goal formation” [5], and the essence of “personal choice” [6].

In general, as emphasised by many scholars researching the processes of the regulation of a person’s life, the problems of phenomenology, forms, and mechanisms of semantic regulation processes and organisation of human life are still underdeveloped. Theoretical schemes of regulation prevailing in science cannot reveal the regularities of the processes focused on fuzzy and blurred states and criteria that are re-determined and changed over the course of life. This is especially true in regard to the problems of creative longevity in teaching and the value-semantic regulation of the life of a teacher as the subject of his own life path at the final stages of professionalisation.

The basis of the approaches regarding the analysis of the above-mentioned problems should be formed by psychological concepts reflecting the laws of the entire individual life path. One such concept is the integrative approach developed by E.Y.Korzhova [7]. The attractiveness of this approach for our research is due to its focus on the study of a person as a multidimensional space of meanings and value orientations. Within the integrative approach, a key role in the person’s life path is given to life orientations. It is the choice of life orientations that is understood as the determining moment of a person’s life, including its final stages. An important point is also that the theoretical position of the author is supported by empirical material and, in particular, the developed methodological apparatus that allows us to distinguish different types of life orientation. A significant conceptual contribution to the justification of value-semantic regulation of a teacher’s life as the subject of his life path was made by D.Leont’ev [4] who devoted a number of fundamental studies to the development of general psychological ideas of the human existence semantic dimension. He suggested the concept of semantic regulation of life, based on a common understanding of a person’s semantic structures as transformed forms of life relations. His analysis of the phenomenon of semantic regulation as a constitutive function of a personality shows that “personality as a psychological unit and as a regulatory system is constituted by the functions of a person isolating himself from the surrounding world, presentation and structuring of his relations with the world, and his life activity subordination to the stable structure of these relations as opposed to immediate impulses and external stimuli” [8]. To provide better understanding of the nature of semantic regulation, the author suggests that it is important to consider the question: Why do people do what they do? The possible answer “I did it because it was important for me” is within the logic of meaning and the logic of vital necessity [8]. Meaning-oriented action defines behaviour that takes into account the entire system of a person’s relations with the world and the entire long-term temporal perspective, but a teacher’s long-term pedagogical activity inevitably leads to the collision with a number of negative factors, occupational hazards, and costs. A lot of scholars include the following points into the list of above-mentioned factors. The mentioned factors are as follows: (1) High levels of responsibility for the life and health of children and adolescents, (2) low salary levels in comparison with other branches of the national economy, (3) constant psychological pressure on teachers, both from parents and from the administration of the institution, (4) inevitably arising conflict situations, and (5) emotional exhaustion and emotional burnout. For example, it was found that 7% of modern teachers have emotional exhaustion, 75% suffer from depersonalisation, and 13% of urban school teachers demonstrate emotional burnout [9]. Despite the constant increase in the number of studies of the problem of professional burnout, the question remains why some people develop this syndrome while others do not (provided that they both work in the same organisation and have the same profession). Attempts to explain this by age differences do not provide an unambiguous answer. The same situation is true regarding years of professional experience. Only minor tendencies were found that relate years of professional experience and professional burnout. Besides, there is also a correlation between the degree of career satisfaction and low degrees of work effectiveness [10]. Nevertheless, it is the reality of modern education that there exist “centenarians” in the teaching profession.

Thus, the problem of studying values of activity at the final stages of professionalisation is still kept outside the field of scientific research of the processes of regulation of a person’s life in the profession by a person of mature age. 

The hypothesis of the study consists of the assumption that the values of activity determine the prospects of a teacher’s professional longevity passing from the external environment to the inner plan of personality. We assume that activity as the subject of research can be analysed not only from the point of view of its structure and content but also as a basis for the system of personal value formation.

The project is aimed at studying the mechanisms of regulation of the professional activity of a teacher on the basis of a dynamic approach that considers “movements of the activity itself and its structural components” [11]. Personal values of the professional activity of a teacher are in the center of attention. Such an aspect of the activity’s consideration fundamentally changes the analysis of the processes of self-regulation of the personality and, accordingly, the activity that it performs.

Besides, considering the phenomenon of professional longevity, it is important to understand the psychological nature of professional self-determination. It is the process of the formation of a person’s attitude to the professional and working environment. A teacher comes to productive longevity in the profession through a long process of professional formation. It is achieved through understanding the professional destination, the realisation of their possibilities and the assessment of both current and potential professional abilities. Besides, by overcoming difficult situations in professional activity, a teacher is convinced of his professional compliance.

Some components of a person’s individuality can be acknowledged as professionally significant features of a teacher; they are significant for the success of the professional activity. However, the professional activity of a teacher is characterised by the risks of emotional and personal distress and the lack of appropriate financial compensation. To continue professional activity in such conditions, it is necessary to have a special understanding since the regulatory mechanisms of activity relevant to the previous stages of professional development obviously lose their significance.

We assume that the prospect of professional longevity arises in the process of the teachers’ searching for new value bases of their activities.

In order to identify the value mechanisms that contribute to the long-term effective functioning of the individual within the teaching profession, an attempt has been made to justify the criteria of professional longevity, ensuring the efficiency, reliability, and productivity of the teacher. 

For this purpose, we analyse the methods and methodologies which allow us to hypothesise and carry out a practical pilot study to identify significant relationships of professional longevity with some psychological factors of a value-semantic nature.

## 2. Materials and Methods

Two hundred and eighty teachers of Volgograd and the Volgograd region participated in the research. The average age of the study participants was 44.3 years. The number of men was less than 5% of the sample. This reflects the composition of teachers in Russia’s educational institutions nowadays. Ninety percent of subjects have a higher education. The average experience of teachers was 19.8 years. The average experience of teachers in the institution examined by us was 13.8 years. This fact indicates the importance of workplace stability for Russian educators.

During preparatory work, the test battery, which included the following techniques, was developed:

1. The technique of “Psychological portrait of the teacher”, in our opinion, contains high diagnostic potential as it allows the estimation of the orientation of the identity of the teacher as a professional depending on acceptance of profession values, emotional conditions, self-assessment levels, styles of teaching, levels of subjective control, and satisfaction with work. This technique is widely used in research on Russian-speaking teachers [12].
2. The Russian adaptation of the General Self-Efficacy scale [13,14]. The scale of self-efficiency determines, with a sufficient degree of validity, the level of self-effectiveness of the person in accordance with the understanding of the term proposed in the concept of A. Bandura [15]. This express scale has high levels of reliability and validity, as well as easily fitting into the overall orientation of the study. The use of this scale as the behavioural indicator was assumed. The role of self-efficacy as the basic conviction of the individual in his own success, readiness, and ability to overcome any obstacles in the context of our research is as follows:we assume that the high level of self-efficiency is one of the conditions that ensure the functioning of the value-meanings mechanisms of the person;that is, the high level of self-effectiveness does not belong to the content of the value-meanings sphere, however, it is necessary for the person as an obligatory condition of continuation of professional activity in the post-retirement period.

On the one hand, the technique was included in the battery testing as an additional scale, which determines the behavioural component of the current situation of professional development. On the other hand, the value-meaning regulation of activity is inconceivable without connection with readiness to the realisation of set purposes, the achievement of the intended values. We consider excessive enumeration of research which repeatedly confirm the interrelation of levels of self-effectiveness with the real achievements of the person—e.g., educational, sports, professional.

3. The modification of a technique of R. Inglehart for studying values of the structure of mass consciousness [16]. The value of this approach for our research was assumed, proceeding from the conceptual position of the author about natural “shift” of values in the process of social progress from materialistic to individualisation values [17].
4. The technique “Meaning of life orientation” (SJO-Russian version)is an adapted version of the Purpose-in-Life test (PIL) test [6]. This technique is the most quoted and used in various basic and applied research in Russia. Its use in our work is dictated by the aspiration to provide validity of the obtained data and also to give an opportunity to other researchers to compare our results to others.
5. The technique of the diagnostics of emotional burnout was included in the testing battery; on the one hand, as an indicator of psychophysical health, and on the other hand, in connection with the assumption that one of the basic indicators of professional longevity in the teaching profession should be the ability of the teacher to cope with the symptoms of the syndrome at various levels, including, at the level of sense formation, the generation of new meanings in difficult conditions of activity, including in conditions of accumulating stress. This technique has high validity, which is confirmed by numerous experimental data and also our sample [10].

We also used several other techniques.

## 3. Results

### 3.1. Results on a Technique Self-efficiency Scale (GSES)

The data obtained by us practically correspond to the statistics published by authors who have used this scale since its publication. The data of descriptive statistics, including the use of strict parametric methods of checking statistical hypotheses of differences on separate age groups, lead to conclusions about insignificant corrections of the average score.

**Figure 1:**
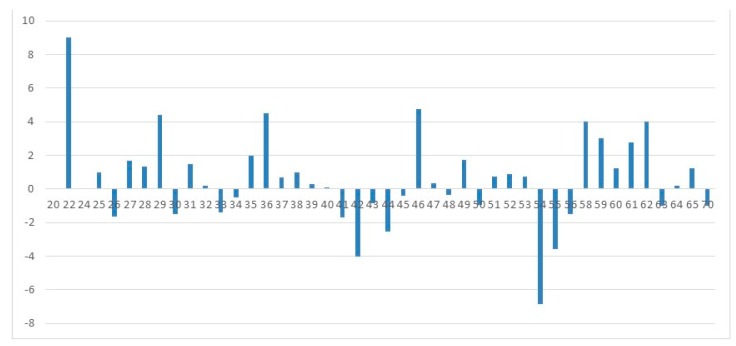
Deviations from an arithmetic average of the general indicator General Self-Efficacy scale (GSES) by Schwarzer-Jerusalem’s technique, depending on age [7].

However, if we consider the data on the temporary deployment, in particular in terms of age and general pedagogical experience of respondents (see Figure 1 below), we can say that there are a number of trends.

The presence of wave-like dynamics of the indicator of the CE and peak “ages”—both ascending and descending.

Accordingly, it is possible to allocate time periods—“positive phases” to “negative phases”.

Special attention should be paid to the pronounced dynamics in the area of pre-retirement age and the beginning of retirement age.

Let us stop in detail on the last highlighted point. We see, on the one hand, a sharp decline in self-confidence among teachers of 54–56 years, and then, in the 58–62 year range, there is a long period of high self-efficacy. In our opinion, this picture can be explained by teachers with a high level of self-effectiveness in educational institutions remaining to work during the pension period. Therefore, our assumption is confirmed.

### 3.2. Results According to Method PIL (Adapted by D. A. Leontiev, Russian Version-SJO)

**Figure 2:**
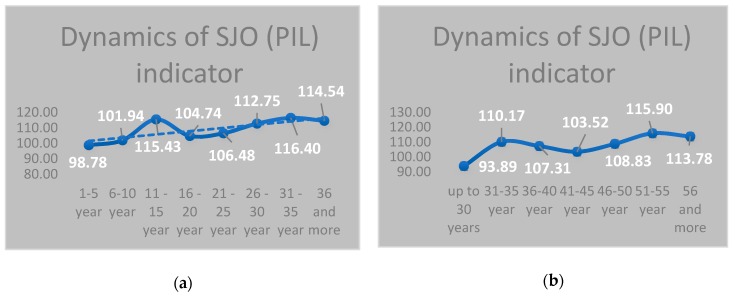
The dynamics of SJO (Purpose-in-Life test (PIL)) indicator on ordinate axis–seniority size (**a**); age (**b**).

In Figure 2, the dynamics of an indicator of SJO (PIL) for teachers of different age (a) and length of service(b) is presented. The general trend is an increase in GPA of SJO in the process of increase in age and length of service. This fact meets the expectations of the Soviet and Russian psychologists of the importance of processes of a meaningful development in personal life. 

In Figure 2a, we see two ascending peaks (31–35 and 51–55 years) and one descending peak (41–45 years). In both drawings, the accurate ascending of the LSS trend is observed, and in the age period up to 30 years, it is less reliable than at other ages.

The analysis SJO (PIL) dynamics depending on the seniority of respondents (Figure 2b). We see a similar picture—two peaks (the experience of 11–15 years and 31–35 years). In our opinion, it is an indicator of the classical “professional growth”—the achievement of the highest point of development, i.e., “acme”—with the inevitable subsequent recession (“crisis”).

Along with it is also worth noting the interrelations revealed by us between separate indicators and an indicator by LSS technique (see Table 1, Table 2 and Table 3). These data convincingly speak about the interrelation between valuable and semantic regulation and vital achievements of teachers.

### 3.3. Results on a Technique by C. Maslach

**Figure 3:**
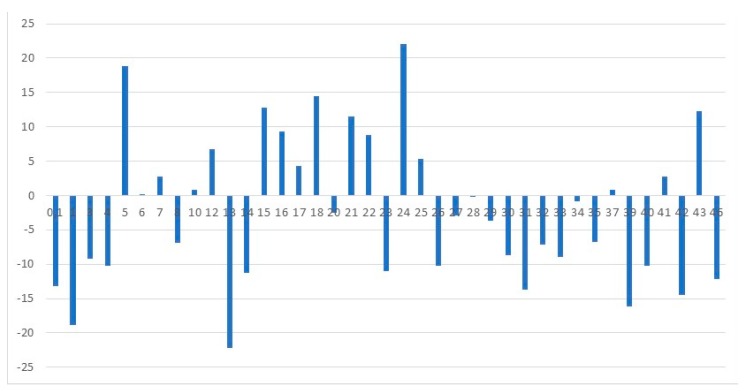
Deviations from an arithmetic average of the general indicator by the C. Maslach technique (adapted by N.E. Vodopyanova [10] depending on the length of service).

We can note that by a technique, teachers up to 50 years have higher points. Teachers who are more senior than 50 years, as a rule, have low indicators on this scale. Proceeding from our data, it can be said that for a stage of professional longevity, the lack of symptoms of emotional burning out is typical. It is that such teachers have the ability to resist adverse factors of the environment. We believe, taking into account the above-stated data, that a key role in the technique is played by the meaning of a coping mechanism (Figure 3).

### 3.4. Analysis of Differences between the Educational Organisations

The modern researchers studying organisational culture agree in opinion on a large variety of definitions of this phenomenon, and it can be noted that organisational culture is what distinguishes one organisation from another even within a formal control system. Within our research, differences that allow teachers to keep professional longevity at some schools longer than in others are most important. We find it possible to assume that the coincidence of personal ideas of self-development in the professional sphere with the requirements of the organisation gives a chance to the worker to be more self-assured and, with positive changes, effective in work. Let us address the obtained data.

Teachers from four schools took part in our study:

(1) School A is located on the outskirts of the city and is undergoing a process of organisational restructuring;
(2) School B is located in the “working” area of the city and has the longest history among the institutions examined;
(3) School C is located in the city center and is one of the best educational institutions in Russia;
(4) School D is located in a remote area of the Volgograd region.

We see in Table 4 that a higher point of self-efficiency comes to light for the teachers of schools A and B. However, indicators by other techniques convincingly say that at school B, there are signs of optimum organisational culture; in particular, signs of favourable motivational climate are observed (Table 5 and Table 6).

The general level of satisfaction with work substantially is defined by a similar vision of the purposes of professional activity between employees and management (Table 6).

High level of orientation to pupils somewhat also reflects orientation to the qualitative, but not quantitative, indices in work that, according to our hypothesis, is an important sign of the organisational culture promoting the professional longevity of teachers (Table 7).

## 4. Discussion and Conclusions

We have a number of the facts which confirm a hypothesis that valuable and semantic mechanisms are the basis of the creation of the professional careers of teachers of “mature age”. The prospect of professional longevity arises for teachers during the search for new valuable bases of activity by them which proceeds in intense conditions, bears in itself risks of emotional and personal trouble and is characterised by lack of the corresponding material remuneration. To continue professional activity in such conditions, it is necessary to conduct special judgment as to the regulatory mechanism of activity that is relevant to the previous stages of professional development. On the basis of the obtained data, it is possible to conclude that the valuable and semantic nature of professional longevity is closely connected with such indicators as the activities of a person in different life situations, increase of intelligence, shift of valuable orientation from life support values to the value of self-realisation and individualisation (as orientation to disclosure in the teaching profession).

Other indicators of valuable and semantic regulation of the teachers focused on the continuation of their professional careers during their retirement period are an increase of the work satisfaction level and an increase in attention to the strengthening and preservation of psychophysical health at the final stages of their professional development. 

In this context it is worth paying attention to a number of facts, among which are the following: (1) teachers of a retirement age show higher level of somatic health than teachers of middle age, and(2) teachers of a retirement age show a stable reduction of symptoms of emotional burnout in comparison with middle-aged teachers. It demonstrates the changes of a valuable and semantic dominant in the course of the activation of the professional resources defining the efficiency of professional activity at this age.

The analysis of dynamics of the studied data shows essential differences between actual age dynamics depending on the teacher’s natural age and dynamics caused by general seniority. If in age dynamics, the signs of so-called “middle age” crisis (40–45 years) can be clearly seen, analysis of the professional age demonstrates stable signs of professional longevity. Thus, the phenomenon of professional longevity is caused more by the processes of an individual’s professional formation than the fact of achieving a certain age. 

The essential factor influencing mechanisms of valuable and semantic regulation of professional longevity are features of pedagogical activity in relation to the level of the educational organisation. There are certain differences between teachers of comprehensive high schools and preschool educational institutions. Analysis of these distinctions allows us to come to the conclusion that a particular culture of an educational institution and/or obstacles play great role in providing valuable and semantic regulation of professional longevity.

Analysis of obtained data and their comparison to basic provisions of the theory of E. Shane [18] about the catalysts and inhibitors of organisational changes allow us to assume that the culture of an organisation is very important and it contributes to the development of employees in terms of the extension of their professional longevity.

Such organisational culture has the following signs:

- goodwill in management relations of employees among themselves;
- the coincidence of the teachers’ personal representations connected with self-development in the professional sphere and complying with the requirements of the organisation;
- existence of joint activity and exchange of experience in the professional sphere (unfinished);
- a similar vision of the purposes of professional activity among employees;
- low level of individual competitive spirit among teachers;
- orientation to a large extent to qualitative, but not quantitative, indices in work.

Based on the data obtained, it can be concluded that the value-semantic nature of professional longevity is closely connected with indicators such as the activation of personal position in life situations, an increase of life meaningfulness, a shift in the value orientations from life support values to the values of self-realisation and individualisation (as an orientation toward disclosure) of a person in the teaching profession.

## Figures and Tables

**Table 1:** Results of the SJO(PIL) indicator depending on assessment by the respondents of the financial position.

Self-Assessment of Financial Position	SJO-Indicator	Purposes in Life	Process of Life	Result of Life	Locus-Ego	Locus-Life
Excellent-great	113.2	35.3	31.9	26.1	23.4	35.5
Medium	109.7	35.7	31.4	25.3	22.3	32.9
Belowanaverage	105.1	34.2	29.8	24.4	21.9	30.4

**Table 2:** Results of the SJO (PIL) indicator depending on the presence of children.

Children	SJO-Indicator	Purposes in Life	Process of Life	Result of Life	Locus-Ego	Locus-Life
No (no answer)	100.7	32.5	28.0	23.0	20.9	30.3
1 child (I have)	107.6	35.4	30.7	25.0	22.8	31.5
2–3 children	114.1	36.5	32.8	26.4	23.3	34.4

**Table 3:** Results of the SJO (PIL) indicator depending on satisfaction with work.

Satisfaction with Work	SJO-Indicator	Purposes in Life	Process of Life	Result of Life	Locus-Ego	Locus-Life
Low	103.0	34.1	29.1	23.5	21.4	30.2
High	114.4	36.3	32.8	26.8	23.8	34.6

**Table 4:** Results of inspection of teachers by the Self-Efficiency scale (Schwarzer—Erusalem, the Russian modification [14]).

Schools	School A	School B	School C	School D
GPA	29.91	28.68	28.14	27.71
Median	29	28.5	27	27.5
Minimum	24	21	23	12
Maximum	38	40	38	39

**Table 5:** Value of age, length of service in this establishment and the general length of service.

Schools	School A	School B	School C	School D
Age	44.6857	46.2368	45.0952	46
Length of service in this establishment	12.286	17.079	18.476	14.654
General pedagogical experience	21.029	22.105	22.095	23.432

**Table 6:** Degree of satisfaction with work.

Schools	School A	School B	School C	School D
High satisfaction	5.59	5.79	5	5.32
Insufficient satisfaction	3.74	3.71	4	3.71
Lack of satisfaction, adaptation problem	0.68	0.5	1	0.96

**Table 7:** Priority values.

Schools	School A	School B	School C	School D
Orientation to pupils	2.89	3.24	3.14	3.21
Orientation to collective	2.49	2.92	2.71	2.21
Self-targeting	4.63	3.84	4.14	4.57
